# Comparisons of Blood Parameters, Red Blood Cell Deformability and Circulating Nitric Oxide Between Males and Females Considering Hormonal Contraception: A Longitudinal Gender Study

**DOI:** 10.3389/fphys.2018.01835

**Published:** 2018-12-19

**Authors:** Marijke Grau, John Maxwell Cremer, Steffen Schmeichel, Markus Kunkel, Wilhelm Bloch

**Affiliations:** Molecular and Cellular Sports Medicine, German Sport University Cologne, Cologne, Germany

**Keywords:** red blood cells, red blood cell deformability, nitric oxide, blood parameters, gender, hormonal contraception

## Abstract

Red blood cell (RBC) deformability is an important determinant of the microcirculation. It is influenced by various hematological parameters but also by nitric oxide (NO) which is produced in RBC from L-arginine by RBC-NO synthase. Longitudinal studies on blood profile, deformability at rest and NO levels but also differences between males and females (±hormonal contraception; HC) are less known so far. The study thus aimed to investigate RBC deformability, RBC NO species (nitrite, RxNO), RBC L-arginine concentration and basal blood parameters in males and females (±HC) as a function of time. RBC deformability was measured at rest once per week and the remaining parameters were measured once per month, respectively. A second experiment aimed to daily measure RBC deformability and 17β-estradiol in Female ± HC during a whole menstruation cycle to investigate a possible relation of the two parameters. Measured parameters showed low week-to-week variation and remained constant during study period. However, RBC deformability increased in Female + HC during study period possibly because of increasing training volume of the participants. Overall, results indicate gender differences in hematological parameters with higher RBC parameters (RBC count, hematocrit, hemoglobin concentration) in males compared to females. Differences were also observed between the female groups with Females - HC showing lower number of RBC but higher MCV and hematocrit compared to Females + HC. RBC deformability was highest in Females - HC which might be related to permanent higher estradiol levels and/or higher RBC NO levels because RBC nitrite and RBC RxNO concentrations were also highest in Females-HC. Results of the second experiment also suggest higher RBC deformability in Female - HC because of higher estradiol concentrations. L-arginine levels known to be related to RBC NO production were comparable in all groups. In conclusion, hematological, hemorheological and NO related parameters show gender differences. In particular, RBC deformability is affected by training volume and RBC estradiol concentrations. The results add new information on the complex regulation of RBC function which might help to better understand the role of RBC in the microcirculation.

## Introduction

Red blood cell deformability describes the ability of the cells to reversibly change their shape in response to applied forces. This relevant characteristic enables the passage of RBC through blood vessels with diameters smaller than their own and thus, the supply of oxygen and nutrient to the surroundings (Mohandas and Chasis, 1993). RBC deformability is influenced by various factors with nitric oxide (NO) being one of them (Kleinbongard et al., 2006; Grau et al., 2013). Reduced RBC NO bioavailability has been reported to reduce RBC deformability while increased levels of RBC NO has been shown to positively affect RBC deformability (Bor-Kucukatay et al., 2003; Grau et al., 2013). In RBC, NO is enzymatically produced by RBC-NO synthase (RBC-NOS) during the conversion of the RBC-NOS substrate L-arginine to L-citrulline (Kleinbongard et al., 2006). RBC-NOS activation state depends on the phosphorylation of RBC-NOS serine 1177 residue which is related to upstream activation of the phosphatidylinositol 3 (PI3)-kinase/Akt kinase pathway (Suhr et al., 2012). NO is a highly diffusible inorganic radical and described reaction routes of NO within RBC are diverse (Ozüyaman et al., 2008). The RBC NO pool includes, among others, nitrite as primary NO oxidation product and RxNO (sum of nitrosated and nitrosylated species) (Hendgen-Cotta et al., 2008). Further, it was shown that RBC-NOS produced NO leads to *S*-nitrosylation of cytoskeletal spectrins which, although the exact mechanism remains to be investigated, positively affects RBC deformability (Grau et al., 2013). Changes in RBC-NOS activation and concomitant NO levels were related to changes in deformability (Grau et al., 2013).

Reduced deformability levels have been described for patients suffering from cardiovascular diseases, diabetes mellitus or sickle cell anemia (Ballas, 1991; Keymel et al., 2011), and reduction in deformability has been associated to microcirculatory disturbances (Lau et al., 1995; Cicco and Pirrelli, 1999; Kameneva et al., 1999). Increased deformability levels have been shown for endurance athletes (Smith et al., 1999; Tomschi et al., 2018a) and associated to improved performance capacity (Koliamitra et al., 2017; Tomschi et al., 2018b). Exercise was thus described to positively affect RBC function in both, health and disease (Mairbäurl, 2013; Ahmad et al., 2014; Brinkmann et al., 2016; Koliamitra et al., 2017). In fact, endurance exercise increases shear stresses applied to the RBC which increases RBC-NOS dependent NO production (Suhr et al., 2012) and this was related to increased performance parameters. In most studies, male participants were recruited because hormonal variations of female participants might interfere with the outcome. Thus, less is known about possible differences in RBC deformability between male and female subjects. A study by Kameneva et al. (1999) indicated higher RBC deformability levels in pre-menopausal women while a recent study by Tomschi et al. (2018a) did not show sex differences regarding RBC deformability. This difference might be related to the fact that the use of hormonal contraceptives (HC) was not considered in the studies. HC including birth control pill contain synthetic estrogen and progesterone which prevents ovulation and which elevates the progesterone levels in the body. For these women, a reduction of plasma estradiol levels were reported (Mishell et al., 1972) suggesting that this decrease might relate to reduced RBC deformability (Farber et al., 2018).

The recent literature suggests that the regulation of RBC deformability is far more complex than previously believed and additional information on the regulation of RBC function is needed. All cited studies represent cross-sectional studies while knowledge regarding variation of RBC deformability and associated NO levels from longitudinal studies are missing but would help to understand the stability of RBC NO parameters and RBC deformability. Also, a possible effect of hormonal contraceptives on blood parameters, RBC deformability and related parameters is less known. Respective information would be beneficial to assess the possible impact of HC on RBC functional parameters which might help to further understand the regulation of RBC function. Thus, the present study aimed to examine RBC deformability, RBC NO species (nitrite and RxNO) and RBC L-arginine levels during a 20-week investigation period with a special focus on possible differences between males and females. Additionally, female participants were further divided into two sub-groups depending on the usage of hormonal contraceptives to assess a possible effect of estradiol on RBC function.

## Materials and Methods

A series of experiments was conducted to investigate the study purposes. Experiment 1 aimed to investigate RBC function and related parameters during a 20-week study period in males and females ± HC. Experiment 2 aimed to outline a possible relation between estradiol levels and RBC deformability in females ± HC during one menstruation cycle. The protocols used in this study were approved by the ethics committee of the German Sport University Cologne. These protocols align with the Declaration of Helsinki and all participants gave written informed consent to participate in this study.

**Table 1:** Anthropometric data of the study groups.

	Female + HC	Female - HC	Male
Age [years]	26.0 (4.3)	27.3 (7.3)	24.5 (2.9)
Height [m]	1.71 (0.07)	1.65 (0.04)	1.83 (0.06)
Weight [kg]	67.3 (12.8)	60.5 (7.9)	82.2 (8.5)

*Data are presented as Mean (SD).*

### Experiment 1

#### Participants

Twenty-seven healthy moderately trained subjects (15m/12f: 6 f + HC and 6 f-HC) were recruited. All subjects had a west European background, were non-smokers, and non-blood donors. Hormonal contraceptives used by the female volunteers were oral contraceptive pills consisting of a combination of ethinylestradiol and a member of the gestagen family:

Yasminelle [(Jenapharm GmbH & Co., KG, Jena, Germany): Drospirenon 3 mg/Ethinylestradiol 0.02 mg per pill/]; Chariva [(Gedeon Richter Pharma GmbH, Cologne, Germany): Chlormadinone acetate 2 mg/Ethinylestradiol 0.03 mg per pill]; Daylette [(EMRA-MED Arzneimittel GmbH, Trittau, Germany): Drospirenon 3 mg/Ethinylestradiol 0.02 mg per pill]; MAXIM [(Jenapharm GmbH & Co., KG, Jena, Germany): Dienogest 2 mg/Ethinylestradiol 0.03 mg per pill]; Valette (Jenapharm GmbH & Co., KG, Jena, Germany): Diogenest 2 mg/Ethinylestradiol 0.03 mg per pill. Length of menstrual cycle of Female + HC was 28 ± 0 days and of Female - HC was 28 ± 3.5 days.

**FIGURE 1:**
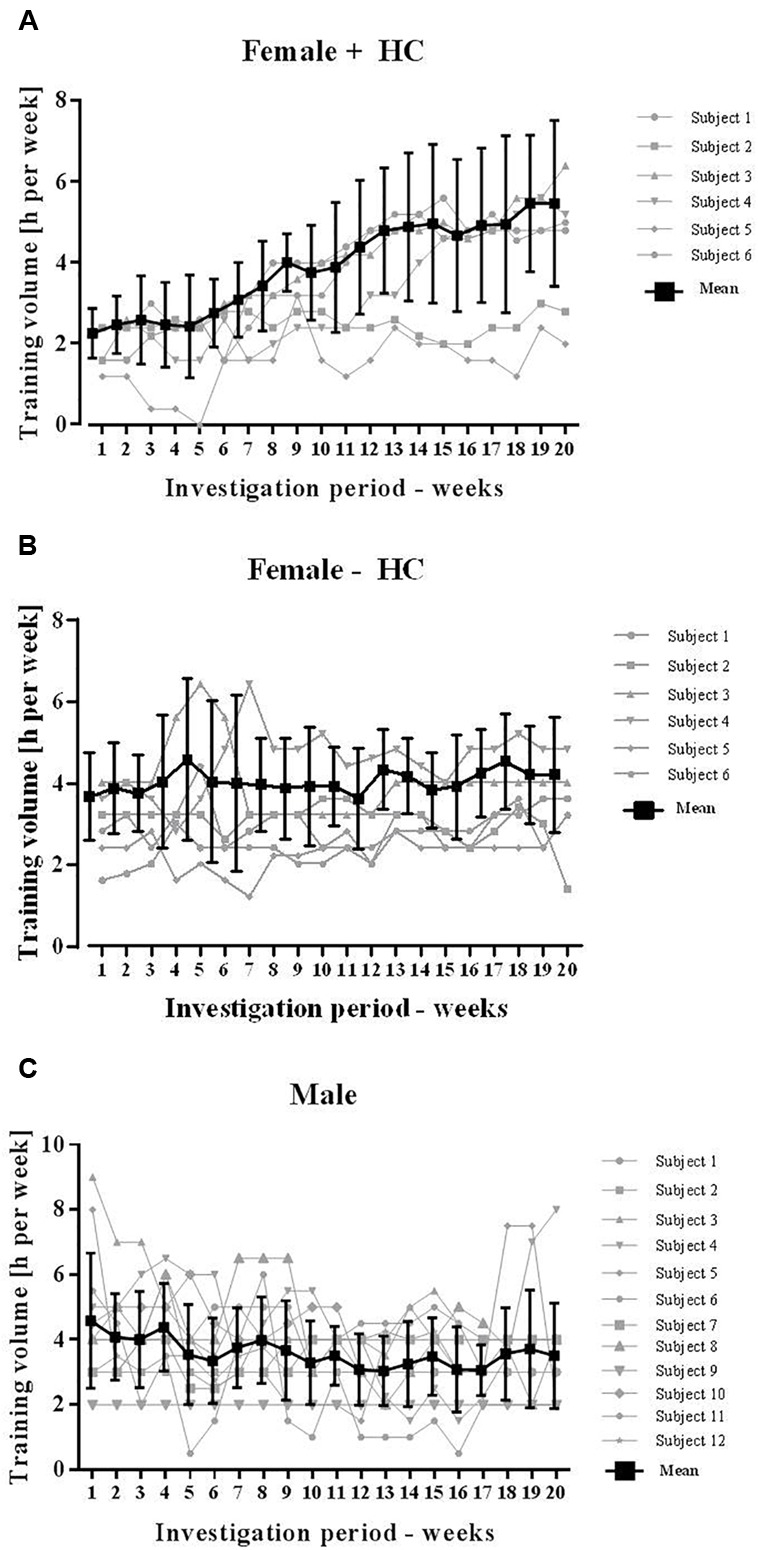
Training volume of participants during study period. Total training volume was recorded on a weekly basis and included both, resistance and endurance training. **(A)** Training hours significantly increased in Female + HC during study period and high variation was observed between the study subjects. **(B)** Training of Female – HC was comparable during the study period. Training volume of Female – HC was higher compared to Female + HC but difference was not statistically significant (*p* = 0.21). **(C)** Training volume of Male was comparable during the study period and lower compared to the female groups, respectively. Difference was not statistically different (Male vs. Female – HC: *p* = 0.14; Male vs. Female + HC: *p* = 0.3).

Basal anthropometric parameters of the subjects are presented in Table 1. Average training volume per week was 4.5 ± 0.45 h/week of Female-HC, 3.9 ± 0.6 h/week of Female + HC and 3.6 ± 1.0 h/week of male participants. Because exercise might have an effect on RBC deformability, detailed course of training volume per week was monitored and presented for each group in Figures 1A–C. Blood sampling of all participants was scheduled at the same time starting in January (week 1) and completed in May (week 20) to avoid a possible influence of seasonal changes on blood count parameters which were reported to be pronounced between spring and summer time (Kristal-Boneh et al., 1993).

#### Study Design

Capillary blood was sampled form the finger pulp into sodium heparinized capillaries (EKF Diagnostic, Germany) once per week for a total of 20 weeks to measure RBC deformability as described below. Additionally, venous blood was sampled weeks 1, 4, 7, 11, 14, 17, and 20 to measure blood parameters, RBC nitrite, RxNO, and RBC L-arginine, respectively. Participants were scheduled for blood sampling in an overnight fasting state and on the same day of the week and at the same time of day every week, respectively. Physical activities including exercise were prohibited 48 h prior to blood sampling. Female participants started with the study on day 1 of their menstrual cycle. Upon arrival to the laboratory, subjects were asked to rest in supine position for 15 min prior to blood sampling. Capillary blood was sampled immediately processed as described below. At respective weeks, venous blood was sampled from the antecubital vein either into sodium heparin vacutainer (BD, United States) for measurement of NO species (nitrite, RxNO: the sum of all nitroso compounds) and L-arginine, or into EDTA vacutainer (BD) to determine blood parameters as described below. Sodium heparin anticoagulated blood was immediately separated at 5,000 g for 1 min at 4°C. Plasma was discarded. RBC for L-arginine and RxNO measurements were transferred to respective reaction tubes, snap frozen and stored at -80°C until measurement. For RBC nitrite measurements, RBC were mixed with a preservation solution (0.8 mol/l ferricyanide, 0.1 mol/l *N*-ethylmaleimide and 10% Igepal) to preserve RBC nitrite and stored at -80°C until nitrite measurement according to Pelletier et al. (2006).

#### Sample Analysis

##### Blood parameters

Numbers of red blood cells (RBC) [^∗^10^6^/μl], white blood cells (WBC) [^∗^10^3^/μl] and platelets (PLT) [10^3^/μl], hematocrit [%], hemoglobin concentration [g/dl] and mean cellular volume (MCV) [fl] were determined using hematology analyzer Sysmex Digitana KX-21N (Sysmex, Switzerland).

##### Red blood cell deformability

Red blood cell deformability was measured using the Laser-assisted-optical-rotational cell analyser (LORCA; RR Mechatronics, Netherlands) as previously described (Baskurt et al., 2009) and data were analyzed according to the guidelines of Baskurt et al. (2009). Briefly, blood was added to a polyvinylpyrrolidone solution in a 1:250 ratio (PVP; 28 cP, RR Mechatronics) and sheared in a Couette system. The samples were exposed to nine shear stresses between 0.3 and 50 Pa. The shear stresses were consecutively applied to the samples and the resulting diffraction pattern was analyzed by the computer software resulting in an elongation index (EI). The nine EI were then used to calculate the maximum deformability (EI max).

##### RBC nitrite and RBC RxNO

Measurements of NO species were performed according to published protocols (Pelletier et al., 2006; Hendgen-Cotta et al., 2008).

For nitrite measurements, RBC were placed on ice and methanol (VWR international, Germany) was added to the frozen samples and thoroughly mixed. Samples were centrifuged at 21,000 *g*, for 15 min at 4°C and the supernatant was transferred to clean reaction tubes and placed on ice for measurement. Nitrite levels of the supernatants were determined using an ozone-based chemiluminescence NO detector (CLD 88e, EcoPhysics, Switzerland). Samples were injected into an acidified tri-iodide solution that reduces nitrite to NO gas, which was then measured by its gas-phase chemiluminescent reaction with ozone (Grau et al., 2007). Nitrite levels were measured in triplicate. For RxNO measurement, samples were pre-treated with acidified sulfanilamide solution (Sigma-Aldrich, United States) for 15 min at 4°C in the dark. Total sample volume was injected into the reaction chamber and measured as described above. Data analysis was performed with the Chart FIA software (Ecophysics, Switzerland) to integrate the area under the curve. A standard curve was used to calculate nitrite and RxNO concentration in the samples. Nitrite concentrations of the samples were corrected for nitrite levels of methanol and preservation solution, respectively. RxNO contents were corrected for RxNO levels of sulfanilamide.

##### RBC L-arginine

L-arginine concentration of RBC was measured using L-arginine ELISA Kit (Immundiagnostik, Germany). Frozen RBC were lysed for 20 min in an ultrasound bath. Samples were centrifuged at 10,000 *g* for 5 min and the supernatant was used for the analysis. The results were evaluated using 4-parameter-algorithm with L-arginine concentration being inverse proportional to color development.

### Experiment 2

#### Participants

Twenty healthy moderately trained females (12 Female – HC and 8 Female + HC) were recruited. All females had a west European background, were non-smokers, and non-blood donors. Hormonal contraceptives used by the female volunteers were oral contraceptive pills and similar to the ones described in Experiment 1 “Participants.” Length of menstrual cycle of Female + HC was 28 ± 0 days and of Female - HC was 26.4 ± 3.2 days. Basal anthropometric parameters of Females - HC were: 24.5 ± 4.8 years; 1.7 ± 0.07 m; 62.14 ± 6.6 kg and of Females + HC were: 26.4 ± 3.9 years; 1.71 ± 0.08 m; 67.1 ± 3.8 kg. Average training volume per week was 3.0 ± 1.7 h/week of Female + HC and 2.2 ± 0.9 h/week Female - HC.

#### Study Design and Sample Measurement

Blood sampling was scheduled daily at the same time of day for a whole menstruation cycle. The sampling started on the first day of menstruation. Upon arrival to the laboratory, females were asked to rest in supine position for 15 min prior to blood sampling. Capillary blood was sampled from the fingertip and anticoagulated using sodium heparin. For RBC deformability measurement, blood was diluted with PVP, transferred to the LORCA device and measured as described above. For 17β-estradiol measurement, 110 μl blood was sampled from the fingertip into a heparinized capillary, sealed and centrifuged at 1000 *g* for 1 min. Plasma fraction was stored at -20°C until measurement using the 17β-Estradiol ELISA (IBL, Hamburg, Germany).

#### Statistical Analysis

Statistical analyses of data and representation of data were performed using GraphPad Prism 6 software (United States). Data are expressed as mean ± SD and presented for each investigation day. Experiment 1: Whiskers plot comprising all data of the whole study period was additionally presented. Data were analyzed for differences between the three tested groups and during intervention period using Friedman-Test or Kruskal–Wallis-Test where appropriate. Linear regression analysis was performed to test for a relation between training hours and RBC deformability. For Experiment 2, paired *t*-test was applied to test for differences between the two groups. *P*-values < 0.05 were considered significant.

## Results

The tested parameters were presented for all investigation days of the study period and additionally, data of all investigation days were pooled and presented as Whiskers plot.

### Blood Parameters

**FIGURE 2:**
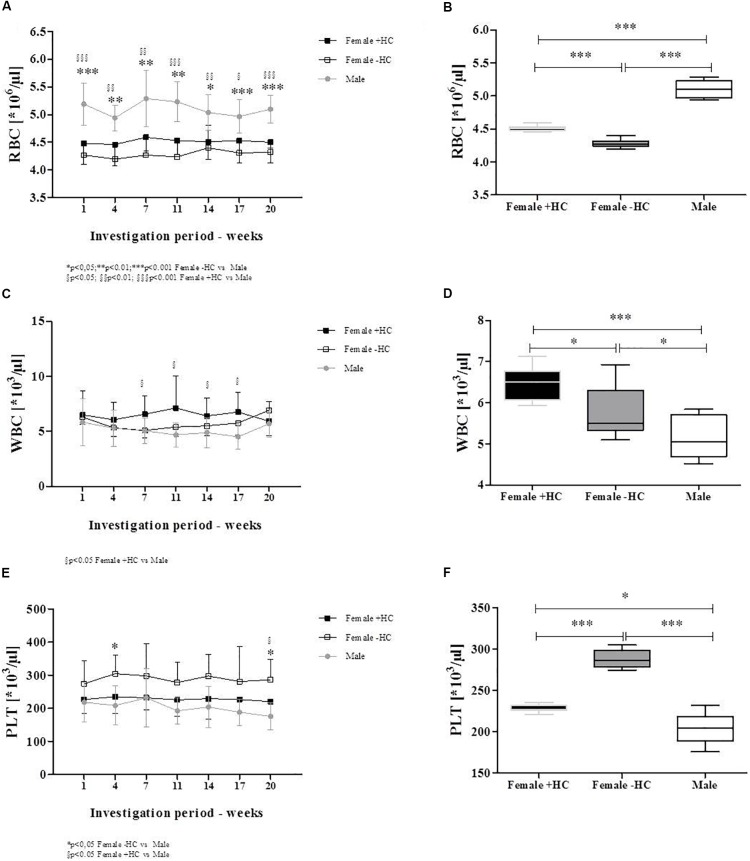
Differences in blood cell count of participants during study period. **(A)** RBC count was highest in Male subjects throughout intervention time which was confirmed by **(B)** Whiskers plot (*p* < 0.001 vs. both Female groups). This further showed higher RBC count in Female + HC compared to Female – HC (*p* < 0.001). **(C)** White blood cell (WBC) count was highest in Female + HC which was also observed for **(D)** summarized data. Female + HC showed higher values compared to Female – HC (*p* < 0.05) and Males (*p* < 0.001). Male subjects showed lowest WBC count. **(E)** Platelet (PLT) count was highest in Female – HC throughout study period as also indicated when **(F)** data were summarized. Female – HC showed higher PLT levels compared to Female – HC (*p* < 0.001) and to Males (*p* < 0.001). Female + HC showed higher values compared to Males (*p* < 0.05).

**FIGURE 3:**
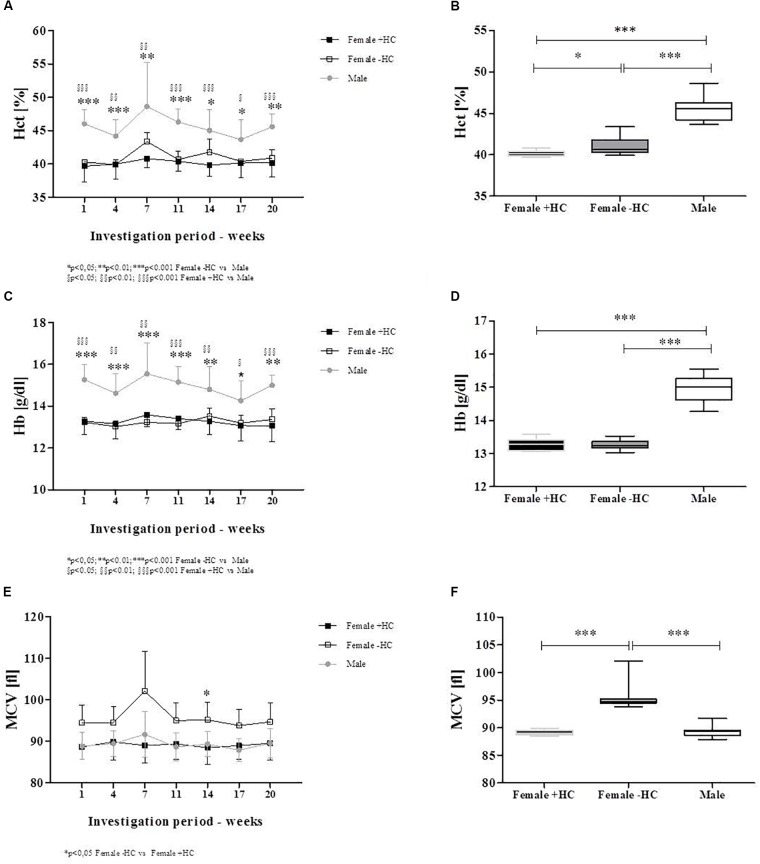
Differences in RBC parameters between participants during intervention. **(A)** Hematocrit levels were highest in Males during the whole study period. **(B)** Whiskers plot presentation revealed significantly higher hematocrit levels in Males compared to Females + HC (*p* < 0.001) and Females – HC (*p* < 0.001). Significant higher hematocrit was measured for Females – HC compared to Females + HC (*p* < 0.05). **(C)** Hemoglobin concentration was highest in Males which was supported by data summary **(D)**. Males showed higher hemoglobin concentration than Female ± HC (*p* < 0.001, respectively). Female groups did not show significantly different values. **(E)** Mean cellular volume (MCV) was highest in Females – HC with **(F)** data summary suggesting significantly higher values in Females – HC compared to Females + HC (*p* < 0.001) and Males (*p* < 0.001), respectively.

Red blood cell count was significantly higher in male participants compared to the two tested female groups for all investigation days (Figure 2A). Summarized data confirmed higher RBC count in Male (*p* < 0.001 vs. Female + HC; *p* < 0.001 vs. Female - HC) and further revealed higher RBC count in Female + HC compared to Female - HC (*p* < 0.001; Figure 2B). White blood cell (WBC) count was highest in Female + HC (Figure 2C) and summarized data indicate significant differences between Female + HC and Female - HC (*p* < 0.05), between Female + HC and Male (*p* < 0.005) and between Female - HC and Male (*p* < 0.05; Figure 2D). Platelet (PLT) count was highest in Female - HC and lowest in Male (Figure 2E). Pooled data underlined higher PLT in Female - HC compared to Female + HC (*p* < 0.01) and Male (*p* < 0^∗^.001), respectively, and lower PLT in Male compared to Female + HC (*p* < 0.05; Figure 2F). Hematocrit values were highest in Male throughout the intervention period (Figure 3A) and data summary showed higher hematocrit levels in Female - HC compared to Female + HC (*p* < 0.05) and higher hematocrit levels in Male compared to female groups (*p* < 0.001, respectively; Figure 3B). Hemoglobin concentration was also higher in Male compared to female groups (Figure 3C). Whiskers plot indicated higher hemoglobin concentration in Male compared to Female + HC (*p* < 0.001) and Female - HC (*p* < 0.001; Figure 3D). Mean cellular volume (MCV) was highest in Female - HC (Figure 3E). Summarized data showed significantly higher values in Female - HC compared to Female - HC (*p* < 0.001) and Male (*p* < 0.001; Figure 3F).

**FIGURE 4:**
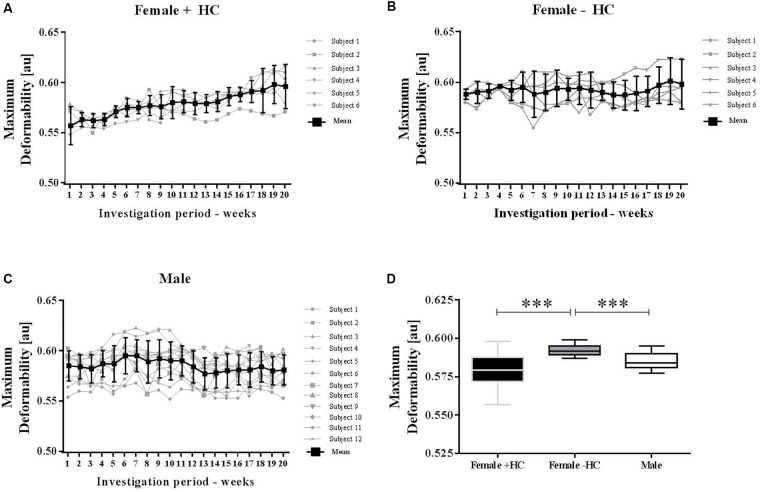
Maximum deformability variation of male and female subjects during investigation period. Individual values were presented along with mean values for **(A)** Female + HC, **(B)** Female – HC, and **(C)** Male participants. Data of all three groups showed low week to week variation but RBC deformability increased with time in Female + HC. **(D)** Maximum deformability was highest in Female – HC compared to Female + HC (^∗∗∗^*p* < 0.001) and compared to Males (*p* < 0.001).

### Red Blood Cell Deformability

#### Experiment 1

Individual RBC deformability data and mean values of the three tested groups were presented in Figure 4. Range of week to week variation was -1.8 to +1.1% for Female + HC; -0.4 to +1.2% for Female - HC and -1.3 to +1.3% for Male. RBC deformability data of weeks 2–20 were compared to RBC deformability data of week 1 and results suggest no difference in RBC deformability in Female - HC and Male participants while in Female + HC significantly higher values were measured weeks 6–20 compared to week 1 (*p* < 0.05 weeks 6–14 and *p* < 0.01 weeks 15–20, respectively; Figures 4A–C). Linear regression between training volume and RBC deformability revealed a moderate positive relation between the tested parameters in Female + HC (*R*^2^ = 0.3563) while no relation was found in Female - HC (*R*^2^= 0.14) and Male (*R*^2^= 0.0314), respectively. Data summary revealed that RBC deformability values were higher in Female - HC compared to Female + HC (*p* < 0.001) and compared to male participants (*p* < 0.001; Figure 4D).

#### Experiment 2

**FIGURE 5:**
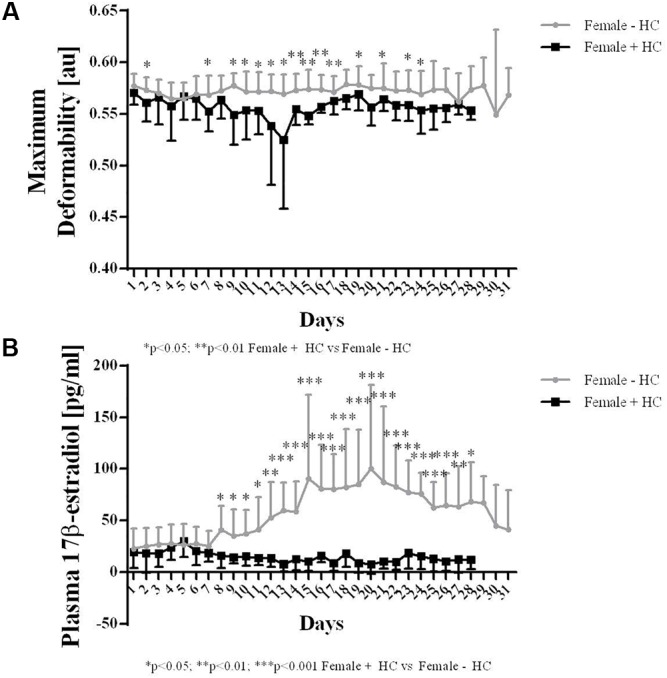
Maximum deformability and 17β-estradiol levels of Females – HC and Females + HC during a menstruation cycle. **(A)** Maximum deformability was higher in Females – HC compared to Females + HC during one menstruation cycle. RBC deformability showed low day to day variation. Cycle length was longer in Female – HC. **(B)** 17β-estradiol levels were comparable throughout the menstruation cycle in Female + HC. In Female – HC, estradiol levels were constant until day 7, then increased with maximum values measured during ovulation and decreased again.

Red blood cell deformability measured during a whole menstruation cycle suggest higher values in Female - HC compared to Female + HC. Significantly higher values were observed in Female - HC on days 2, 7, 9–16, 18, 20, and 22 (Figure 5A).

### 17β-Estradiol Concentration

#### Experiment 2

Hormone concentration of Female + HC was in the range between 8 and 30 pg/ml throughout the whole menstruation cycle while Female - HC showed high estradiol variation ranging from 22 to 40 pg/ml during follicular phase (days 1–10), between 90 and 100 pg/ml during ovulation (days 14–16) and between 60 and 70 pg/ml during mid luteal phase (day 21).

Female - HC showed higher 17β-estradiol levels on days 8–28 compared to Female + HC (Figure 5B).

### RBC Nitrite and RxNO Levels

**FIGURE 6:**
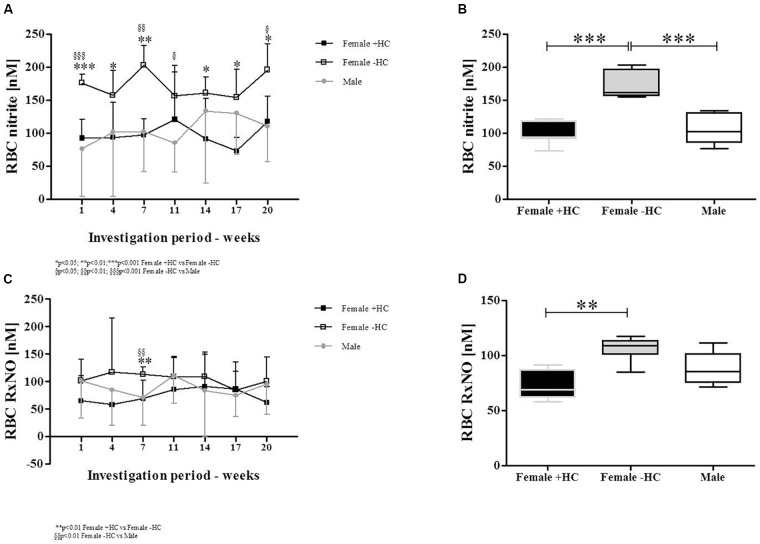
Differences of RBC nitrite and RBC RxNO levels between study subjects during study period. **(A)** RBC nitrite levels were highest in Female – HC throughout the study period. **(B)** Whiskers plot show significantly higher RBC nitrite levels in Female – HC compared to Female + HC (*p* < 0.001) and compared to Males (*p* < 0.001). **(C)** RxNO levels of RBC were higher in Female – HC. **(D)** Significantly higher RxNO values were detected for Females – HC compared to Female + HC (*p* < 0.01) but values of female groups were not significantly different to RxNO levels in Males.

Red blood cell nitrite concentration was highest in Female - HC (Figure 6A). Data summary revealed significantly higher RBC nitrite levels in Female - HC compared to Female + HC (*p* < 0.001) and to Male (*p* < 0.001; Figure 6B). RBC RxNO levels were also highest in Female - HC (Figure 6C). Summarized data revealed significantly higher RBC RxNO levels in Female - HC compared to Female + HC (*p* < 0.01). Difference between Female + HC and Male and Female - HC and Male was not statistically different (*p* > 0.05; Figure 6D).

### L-Arginine Concentration

**FIGURE 7:**
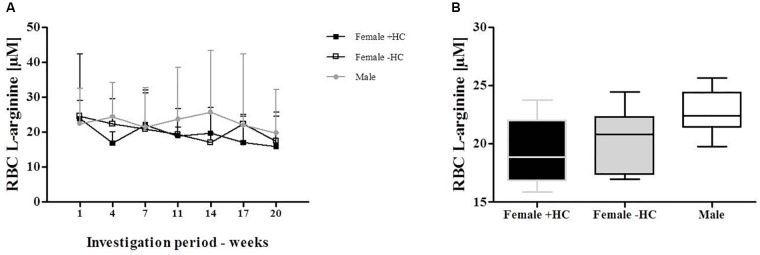
Red blood cell (RBC) L-arginine concentration of Females and Males during investigation period. **(A)** RBC L-arginine levels showed no variation during investigation period and no significant differences between the tested groups. **(B)** This was confirmed by data summary represented as Whiskers plot.

Measured L-arginine levels did not show differences between the tested groups (Figures 7A,B).

## Discussion

The hematological profiles of males and females are well known while only few studies reported gender differences of RBC deformability. Also, less is known about possible variations of blood profile, RBC deformability and related parameters over a longer period of time with a special regard on the influence of hormonal contraceptives on RBC hematological and hemorheological parameters which was thus, aim of the present study.

The key findings of the recent study indicate that blood parameters, RBC deformability and related parameters, such as NO and L-arginine concentration, are stable throughout a long period of time. Week to week variation of RBC deformability for instance was less than 2%. Data were compared between male and female participants and females were further divided into non-HC and HC subgroups. Comparisons of the tested groups indicate differences between males and females but further indicate differences between Females + HC and Females - HC regarding key RBC parameters like RBC count, hemoglobin concentration, hematocrit or MCV, but also RBC deformability and NO parameters were highly different between the female groups and possibly related to differing estradiol concentrations.

### Hematological Parameters

Differences of the blood profile have been already reported between males and females. For instance, Bain (1996) reported higher white blood cell and platelet count in females compared to males. The authors were not able to explain the observed differences but exclude that smoking, diet or hormonal contraceptives have an impact on these parameters (Bain, 1996). The data of the recent study also suggest higher white blood cell and platelet count in women compared to men. In contrast to the finding of Bain (1996) on the effects of HC on white cell count, the data presented herein showed lower WBC count in Female - HC compared to Female + HC. A study of Fisch and Freedman (1975) reported that smoking, obesity and contraceptive use have an impact of WBC count with lower WBC number reported for women without these characteristics. The women included in the present study showed a normal BMI and were non-smokers but were separated according to the information on contraceptive use. It is thus suggested that hormonal contraceptives affect the number of leukocytes but the underlying mechanism remains to be investigated. Another finding of the recent study indicated a higher number of platelets in Female - HC compared to Female + HC. The findings are in accordance to a study by Isaac et al. (2014) who reported lower platelet count in females on long term hormonal contraceptives. Higher number of platelets in Females with higher estradiol levels was also reported by Daly (2011) and explained by a triggering effect of estradiol on proplatelet formation in megakaryocytes.

Differences between men and women were also shown for RBC parameters, i.e., RBC count, hematocrit and hemoglobin concentration. Males show higher hematocrit levels than females (m: 41–50% vs. f: 35–45%) and higher hemoglobin concentrations (m: 13.5–17 g/dl vs. f: 12–16 g/dl) which was associated to higher testosterone levels in men which facilitates erythropoiesis, but also genetic differences in the erythropoietin gene and of its receptor have been reported between males and females (Zeng et al., 2001). Further, women of reproductive age have periodic menstrual blood losses which were reported to reduce hematocrit. Moreover, the results indicate higher hematocrit despite lower RBC number in females who do not take hormonal contraceptives compared to women who take contraceptives. This might be related to increased MCV of the RBC in Females - HC because hematocrit measurements might be affected by MCV values (hct = MCV ^∗^ number of RBC).

Week to week comparison of the hematological profile indicate that the described gender differences were consistent throughout the study period thus representing general gender differences.

### RBC Deformability

Red blood cell deformability was described to be of major importance to transit the smallest capillaries for oxygen supply to the tissues, organs or working muscles. To the best of our knowledge, studies on long-term monitoring of RBC deformability are lacking. Thus, the recent investigation is the first to show low week-to-week intra-group variation of RBC deformability, thus suggesting preserved stability in constant situations. Also, variation in RBC deformability of Female - HC and Male during course of study was low while in Female + HC, RBC deformability increased over time. This increase was highly related to increasing training volume observed in this study group. While training hours of Female - HC and Male remained constant throughout the study period, training hours of Female + HC increased from 2.25 ± 0.61 h/week at week 1 to 5.46 ± 2.05 h/week at week 20 (*p* < 0.01). Increasing training hours were observed in four out of six women. The stated training intensities were rather moderate and type of sports included jogging, cycling or swimming. Linear regression analysis revealed a moderate relation of RBC deformability and training volume in Female + HC while no relation was found in Female - HC and Male, respectively. Published data on the effect of exercise on RBC deformability are inconsistent and explained by variation in exercise intensity and volume, study population tested and/or deformability measuring devices applied (Connes et al., 2013). More recent data suggest a beneficial effect of both, acute exercise and chronic training on RBC deformability in healthy and diseased participants (Gürcan et al., 1998; Smith et al., 1999; Suhr et al., 2012; Ahmad et al., 2013, 2014; Koliamitra et al., 2017; Tomschi et al., 2018a). The data presented herein are thus in line with the cited literature indicating that even moderate training might positively affect RBC function. The increase in RBC deformability during training might be associated to an increased number of young and more flexible RBC which positively affects RBC deformability of the whole RBC population (Smith et al., 1999; Brinkmann et al., 2016; Tomschi et al., 2018b). RBC deformability might also be affected by increased NO production during exercise because higher shear stresses observed during physical activity were described to augment phosphorylation and thus activation of the RBC PI3/Akt kinase pathway which subsequently favors the phosphorylation and thus activation of the NO producing RBC-NOS enzyme (Suhr et al., 2012). The resulting increase in RBC NO levels were shown to positively affect deformability (Kleinbongard et al., 2006; Baskurt et al., 2011; Grau et al., 2013) possibly because of *S*-nitrosylation of the cytoskeletal spectrins (Grau et al., 2013). An increase in NO production caused by increasing training hours seems unlikely because measured nitrite and RxNO levels as representatives of NO production, were lower in Female + HC compared to Female - HC and remained unchanged during the study period.

Comparing RBC deformability of the recent investigation between the study groups revealed that values obtained for Females - HC were highest compared to male and Females + HC while values of males and Females + HC show similar results. A recent study by Tomschi et al. (2018a) suggest comparable RBC deformability values between adolescent males and females but differences in deformability between male and females during transition to adulthood. It was suggested that the onset of sex hormones might affect the RBC system. In the aforementioned study, the use of hormonal contraceptives and phase of menstrual cycle was not taken into account. A study by Kameneva et al. (1999) reported higher deformability of pre-menopausal women compared to age-matched men which was explained by younger RBC population in females due to regular physiologic bleeding. Guillet et al. (1998) reported an influence of sex and menstrual cycle on RBC deformability but no effect of hormonal contraception on RBC deformability. On the contrary, Derham and Buchan (1989) describe a reduction in RBC deformability after taking synthetic progesterons which negatively affected blood viscosity and thus might promote occlusive arterial diseases.

The recent data also indicate an influence of hormonal contraception on RBC deformability. Female participants are often excluded from investigations because hormonal variations during the menstrual cycle were thought to impede the interpretation of the data and might explain varying conclusions from different studies. Serum estradiol is the most potent endogenous estrogen and estradiol levels of women taking hormonal contraceptives was shown to be in the range of 20–30 pg/ml throughout the month (Mishell et al., 1972) while estradiol levels of non-pill users show higher overall values but also high variation with a daily production rate of estradiol being 36 μg during early follicular phase, 380 μg during preovulatory and 250 μg during mid luteal phase (see Reed and Carr, 2015). To further address the question whether estradiol influences RBC deformability, blood samples were taken daily from females with/out contraceptive use during a whole menstruation cycle to investigate and relate the two parameters. Estradiol levels of the recent study support the abovementioned findings of low hormone variation and overall low concentration in Female + HC while hormone status showed high variation during the menstruation cycle in Female - HC with overall higher estradiol levels compared to contraceptive users. RBC deformability was higher in Female - HC but values remained constant throughout the study period suggesting that acute estradiol changes as appear during menstruation cycle do not acutely affect RBC deformability. Instead a general effect of higher estradiol levels on RBC deformability is suggested. A recent study by Farber et al. (2018) also indicate a positive relation between RBC deformability and estradiol levels. RBC possess estrogen receptor α and β but little is known about the estrogen/estradiol pathway within RBC. Unfer et al. (2013) showed that estradiol increases the activity of RBC CuZn superoxide dismutase (SOD). CuZnSOD prevents the formation of cytotoxic oxygen-derived free radicals through the rapid conversion of superoxide anion to hydrogen peroxide (see for review Zelko et al., 2002) and increased activity might enhance antioxidative capacity of the cells. Improved antioxidant capacity might positively affect RBC deformability, especially in situations with higher levels of reactive oxygen/nitrogen species (ROS/RNS) because free radicals were known to decrease RBC deformability (Simmonds et al., 2011). The cohesion of high estradiol levels and higher deformability levels might also be explained by the circumstance that estradiol was thought to increase NO production in endothelial cells (Rubanyi et al., 1997; Haynes et al., 2000) through PI3-kinase-Akt (Haynes et al., 2000) and eNOS activation (Nevzati et al., 2015). Given the fact that endothelial and red blood cell NO production show high similarities (Kleinbongard et al., 2006; Ozüyaman et al., 2008), it is assumed that also in RBC, estradiol might activate NO production through PI3-kinase/Akt kinase/RBC-NOS pathway. Within RBC, NO reaction routes are various. Nitrite has been described as primary oxidation product of NO, thus representing a sensitive marker for NO synthesis (Lauer et al., 2001; Kleinbongard et al., 2006). NO can further react to form nitrosated and nitrosylated NO species which are here summarized as RxNO. The results of the present study revealed that in RBC, nitrite and RxNO levels were higher in Females - HC compared to Females + HC but also compared to the male participants. This might suggest that higher estradiol levels of Females - HC activate NO production in RBC leading to higher concentrations of NO species and thus explaining higher RBC deformability values observed for Females - HC. L-arginine represents the RBC-NOS substrate needed for the conversion of L-arginine to L-citrulline and NO. Levels of RBC L-arginine are approximately around 30 μM in healthy people (Grau et al., 2015) and thus exceed the *K*_m_ reported for the NOS enzyme (2–20 μM) (Eligini et al., 2013). Mean RBC L-arginine levels of the three tested groups ranged between 19.2 and 22.7 μM and thus also exceeded the enzyme *K*_m_. L-arginine levels were comparable throughout the investigation period and levels showed no significant differences between the groups. Thus, differences in the nitrite/RxNO content and of RBC deformability were unlikely to be related to L-arginine availability.

## Conclusion

The results of the present study suggest long-term stability of hematological and hemorheological parameters at constant conditions. Alterations of the regular conditions, such as an increase of weekly training hours, leads to an increase in RBC deformability. Gender differences were observed for RBC dependent blood parameters but also contraceptive use of women was shown to affect the blood profile. Highest RBC deformability data were reported for Female - HC and most likely associated to higher RBC NO production. Differences between the female groups were highly probably related to differing estradiol levels suggested to affect NO synthesizing pathways. Thus, the present results add information on the complex regulation of RBC deformability and recommend to consider gender, training status and hormone levels to interpret basal blood parameters, RBC deformability and related NO data.

## Author Contributions

MG designed the study, collected and analyzed the data, performed statistics, and wrote the manuscript. JC, MK, and SS collected and analyzed the data. WB contributed to study design, interpretation of the data, and the manuscript.

## Conflict of Interest Statement

The authors declare that the research was conducted in the absence of any commercial or financial relationships that could be construed as a potential conflict of interest.

## References

[B1] Ahmad B. Ferrari N. Montiel G. Bloch W. Raabe-Oetker A. Skrobala N. (2013). Influence of a moderate physical activity intervention on red cell deformability in patients suffering from chronic obstructive pulmonary disease (COPD). *Wien. Med. Wochenschr.* 163 334–339. 10.1007/s10354-013-0183-7 23423662

[B2] Ahmad B. Glufke K. Grau M. Sandig D. Rockstroh J. Vogel M. (2014). Influence of endurance training and marathon running on red cell deformability in HIV patients. *Clin. Hemorheol. Microcirc.* 57 355–366. 10.3233/CH-131767 24004552

[B3] Bain B. B. (1996). Ethnic and sex differences in the total and differential white cell count and platelet count. *J. Clin. Pathol.* 49 664–666. 8881919 10.1136/jcp.49.8.664 PMC500612

[B4] Ballas S. K. (1991). Sickle cell anemia with few painful crises is characterized by decreased red cell deformability and increased number of dense cells. *Am. J. Hematol.* 36 122–130. 10.1136/jcp.49.8.664 1707225

[B5] Baskurt O. K. Boynard M. Cokelet G. C. Connes P. Cooke B. M. Forconi S. (2009). New guidelines for hemorheological laboratory techniques. *Clin. Hemorheol. Microcirc.* 42 75–97. 10.1002/ajh.2830360211 19433882

[B6] Baskurt O. K. Ulker P. Meiselman H. J. (2011). Nitric oxide, erythrocytes and exercise. *Clin. Hemorheol. Microcirc.* 49 175–181. 10.3233/CH-2011-1467 22214688

[B7] Bor-Kucukatay M. Wenby R. B. Meiselman H. J. Baskurt O. K. (2003). Effects of nitric oxide on red blood cell deformability. *Am. J. Physiol. Heart Circ. Physiol.* 284 H1577–H1584. 10.1152/ajpheart.00665.2002 12521942

[B8] Brinkmann C. Bizjak D. A. Bischof S. Latsch J. Brixius K. Bloch W. (2016). Endurance training alters enzymatic and rheological properties of red blood cells (RBC) in type 2 diabetic men during in vivo RBC aging. *Clin. Hemorheol. Microcirc.* 63 173–184. 10.3233/CH-151957 26410865

[B9] Cicco G. Pirrelli A. (1999). Red blood cell (RBC) deformability, RBC aggregability and tissue oxygenation in hypertension. *Clin. Hemorheol. Microcirc.* 21 169–177. 10711739

[B10] Connes P. Simmonds M. J. Brun J. F. Baskurt O. K. (2013). Exercise hemorheology: classical data, recent findings and unresolved issues. *Clin. Hemorheol. Microcirc.* 53 187–199. 23042105 10.3233/CH-2012-1643

[B11] Daly M. E. (2011). Determinants of platelet count in humans. *Haematologica* 96 10–13. 10.3324/haematol.2010.035287 21193429 PMC3012758

[B12] Derham R. J. Buchan P. C. (1989). Haemorheological consequences of oestrogen and progestogen therapy. *Eur. J. Obstet. Gynecol. Reprod. Biol.* 32 109–114. 10.1016/0028-2243(89)90191-3 2673883

[B13] Eligini S. Porro B. Lualdi A. Squellerio I. Veglia F. Chiorino E. (2013). Nitric oxide synthetic pathway in red blood cells is impaired in coronary artery disease. *PLoS One* 8:e66945. 10.1371/journal.pone.0066945 23940508 PMC3734222

[B14] Farber P. L. Freitas T. Saldanha C. Silva-Herdade A. S. (2018). Beta-estradiol and ethinylestradiol enhance RBC deformability dependent on their blood concentration. *Clin. Hemorheol. Microcirc.* 70 339–345. 10.3233/CH-180392 29710691

[B15] Fisch I. R. Freedman S. H. (1975). Smoking, oral contraceptives, and obesity. Effects on white blood cell count. *JAMA* 234 500–506. 10.1001/jama.1975.03260180040020 1242167

[B16] Grau M. Friederichs P. Krehan S. Koliamitra C. Suhr F. Bloch W. (2015). Decrease in red blood cell deformability is associated with a reduction in RBC-NOS activation during storage. *Clin. Hemorheol. Microcirc.* 60 215–229. 10.3233/CH-141850 24928922

[B17] Grau M. Hendgen-Cotta U. B. Brouzos P. Drexhage C. Rassaf T. Lauer T. (2007). Recent methodological advances in the analysis of nitrite in the human circulation: nitrite as a biochemical parameter of the L-arginine/NO pathway. *J. Chromatogr. B Analyt. Technol. Biomed. Life Sci.* 851 106–123. 10.1016/j.jchromb.2007.02.002 17344107

[B18] Grau M. Pauly S. Ali J. Walpurgis K. Thevis M. Bloch W. (2013). RBC-NOS-dependent S-nitrosylation of cytoskeletal proteins improves RBC deformability. *PLoS One* 8:e56759. 10.1371/journal.pone.0056759 23424675 PMC3570529

[B19] Guillet R. Driss F. Perrotin P. Pautou C. Nalpas B. Boynard M. (1998). Gender, menstrual cycle, oral contraceptives and red blood cell deformability in healthy adult subjects. *Clin. Hemorheol. Microcirc.* 19 83–88. 9849921

[B20] Gürcan N. Erbas D. Ergen E. Bilgehan A. Dündar S. Aricioglu A. (1998). Changes in blood haemorheological parameters after submaximal exercise in trained and untrained subjects. *Physiol. Res.* 47 23–27. 9708697

[B21] Haynes M. P. Sinha D. Russell K. S. Collinge M. Fulton D. Morales-Ruiz M. (2000). Membrane estrogen receptor engagement activates endothelial nitric oxide synthase via the PI3-kinase-Akt pathway in human endothelial cells. *Circ. Res.* 87 677–682. 10.1161/01.RES.87.8.677 11029403

[B22] Hendgen-Cotta U. Grau M. Rassaf T. Gharini P. Kelm M. Kleinbongard P. (2008). Reductive gas-phase chemiluminescence and flow injection analysis for measurement of the nitric oxide pool in biological matrices. *Methods Enzymol.* 441 295–315. 10.1016/S0076-6879(08)01216-0 18554541

[B23] Isaac I. Z. John R. T. Suleiman A. S. Erhabor O. Ahmed Y. (2014). The effect of hormonal contraceptives on platelet count of women in Sokoto State North Western Nigeria. *Merit. Res. J. Med. Med. Sci.* 2 007–011.

[B24] Kameneva M. V. Watach M. J. Borovetz H. S. (1999). Gender difference in rheologic properties of blood and risk of cardiovascular diseases. *Clin. Hemorheol. Microcirc.* 21 357–363. 10711771

[B25] Keymel S. Heiss C. Kleinbongard P. Kelm M. Lauer T. (2011). Impaired red blood cell deformability in patients with coronary artery disease and diabetes mellitus. *Horm. Metab. Res.* 43 760–765. 10.1055/s-0031-1286325 22009370

[B26] Kleinbongard P. Schulz R. Rassaf T. Lauer T. Dejam A. Jax T. (2006). Red blood cells express a functional endothelial nitric oxide synthase. *Blood* 107 2943–2951. 10.1182/blood-2005-10-3992 16368881

[B27] Koliamitra C. Holtkamp B. Zimmer P. Bloch W. Grau M. (2017). Impact of training volume and intensity on RBC-NOS/NO pathway and endurance capacity. *Biorheology* 54 37–50. 10.3233/BIR-16121 28697553

[B28] Kristal-Boneh E. Froom P. Harari G. Shapiro Y. Green M. S. (1993). Seasonal changes in red blood cell parameters. *Br. J. Haematol.* 85 603–607. 10.1111/j.1365-2141.1993.tb03354.x 8136283

[B29] Lau C. S. Saniabadi A. R. Belch J. J. (1995). Reduced red blood cell deformability in patients with rheumatoid vasculitis. Improvement after *in vitro* treatment with dipyridamole. *Arthritis Rheum.* 38 248–253. 10.1002/art.1780380214 7848316

[B30] Lauer T. Preik M. Rassaf T. Strauer B. E. Deussen A. Feelisch M. (2001). Plasma nitrite rather than nitrate reflects regional endothelial nitric oxide synthase activity but lacks intrinsic vasodilator action. *Proc. Natl. Acad. Sci. U.S.A.* 98 12814–12819. 10.1073/pnas.221381098 11606734 PMC60136

[B31] Mairbäurl H. (2013). Red blood cells in sports: effects of exercise and training on oxygen supply by red blood cells. *Front. Physiol.* 12:332 10.3389/fphys.2013.00332 PMC3824146 24273518

[B32] Mishell D. R. Jr. Thorneycroft I. H. Nakamura R. M. Nagata Y. Stone S. C. (1972). Serum estradiol in women ingesting combination oral contraceptive steroids. *Am. J. Obstet. Gynecol.* 114 923–928. 10.1016/0002-9378(72)90098-1 4645131

[B33] Mohandas N. Chasis J. A. (1993). Red blood cell deformability, membrane material properties and shape: regulation by transmembrane, skeletal and cytosolic proteins and lipids. *Semin. Hematol.* 30 171–192. 8211222

[B34] Nevzati E. Shafighi M. Bakhtian K. D. Treiber H. Fandino J. Fathi A. R. (2015). Estrogen induces nitric oxide production via nitric oxide synthase activation in endothelial cells. *Acta Neurochir. Suppl.* 120 141–145. 10.1007/978-3-319-04981-6_24 25366614

[B35] Ozüyaman B. Grau M. Kelm M. Merx M. W. Kleinbongard P. (2008). RBC NOS: regulatory mechanisms and therapeutic aspects. *Trends Mol. Med.* 14 314–322. 10.1016/j.molmed.2008.05.002 18539530

[B36] Pelletier M. M. Kleinbongard P. Ringwood L. Hito R. Hunter C. J. Schechter A. N. (2006). The measurement of blood and plasma nitrite by chemiluminescence: pitfalls and solutions. *Free Radic. Biol. Med.* 41 541–548. 10.1016/j.freeradbiomed.2006.05.001 16863986

[B37] Reed B. G. Carr B. R. (2015). “The normal menstrual cycle and the control of ovulation,” in *Hormones and Behavior*, eds De Groot L. J. Chrousos G. Dungan K. (South Dartmouth, MA: MD Text.com, Inc.).

[B38] Rubanyi G. M. Freay A. D. Kauser K. Sukovich D. Burton G. Lubahn D. B. (1997). Vascular estrogen receptors and endothelium-derived nitric oxide production in the mouse aorta. Gender difference and effect of estrogen receptor gene disruption. *J. Clin. Invest.* 99 2429–2437. 10.1172/JCI119426 9153286 PMC508083

[B39] Simmonds M. J. Meiselman H. J. Marshall-Gradisnik S. M. Pyne M. Kakanis M. Keane J. (2011). Assessment of oxidant susceptibility of red blood cells in various species based on cell deformability. *Biorheology* 48 293–304. 10.3233/BIR-2012-0599 22433570

[B40] Smith J. A. Martin D. T. Telford R. D. Ballas S. K. (1999). Greater erythrocyte deformability in world-class endurance athletes. *Am. J. Physiol.* 276 H2188–H2193. 10.1152/ajpheart.1999.276.6.H2188 10362703

[B41] Suhr F. Brenig J. Müller R. Behrens H. Bloch W. Grau M. (2012). Moderate exercise promotes human RBC-NOS activity, NO production and deformability through Akt kinase pathway. *PLoS One* 7:e45982. 10.1371/journal.pone.0045982 23049912 PMC3457942

[B42] Tomschi F. Bloch W. Grau M. (2018a). Impact of type of sport, gender and age on red blood cell deformability of elite athletes. *Int. J. Sports Med.* 39 12–20. 10.1055/s-0043-119879 29165733

[B43] Tomschi F. Bizjak D. A. Bloch W. Latsch J. Predel H. G. Grau M. (2018b). Deformability of different red blood cell populations and viscosity of differently trained young men in response to intensive and moderate running. *Clin. Hemorheol. Microcirc.* 69 503–514. 10.3233/CH-189202 29710695

[B44] Unfer T. C. Maurer L. H. Kemerich D. M. Figueiredo C. G. Duarte M. M. Gelain D. P. (2013). Non-genomic, direct modulatory effect of 17β-estradiol, progesterone and their synthetic derivatives on the activity of human erythrocyte CuZn superoxide dismutase. *Free Radic. Res.* 47 219–232. 10.3109/10715762.2012.762770 23297859

[B45] Zelko I. N. Mariani T. J. Folz R. J. (2002). Superoxide dismutase multigene family: a comparison of the CuZn-SOD (SOD1), Mn-SOD (SOD2), and EC-SOD (SOD3) gene structures, evolution, and expression. *Free Radic. Biol. Med.* 33 337–349. 10.1016/S0891-5849(02)00905-X 12126755

[B46] Zeng S. M. Yankowitz J. Widness J. A. Strauss R. G. (2001). Etiology of differences in hematocrit between males and females: sequence-based polymorphisms in erythropoietin and its receptor. *J. Gend. Specif. Med.* 4 35–40. 11324238

